# Lessons from Hippocrates: Time to Change the Cancer Paradigm

**DOI:** 10.1155/2020/4715426

**Published:** 2020-06-07

**Authors:** Carlos M. Galmarini

**Affiliations:** Topazium Artificial Intelligence, Paseo de la Castellana 40, 28046 Madrid, Spain

## Abstract

The ultimate goal of all medical activity is to restore patients to a state of complete physical, mental, and social wellbeing. In cancer, it is assumed that this can only be obtained through the complete eradication of the tumor burden. So far, this strategy has led to a substantial improvement in cancer survival rates. Despite this, more than 9 million people die from cancer every year. Therefore, we need to accept that our current cancer treatment paradigm is obsolete and must be changed. The new paradigm should reflect that cancer is a systemic disease, which affects an individual patient living in a particular social reality, rather than an invading organism or a mere cluster of mutated cells that need to be eradicated. This Hippocratic holistic view will ultimately lead to an improvement in health and wellbeing in cancer patients. They deserve nothing less.

## 1. Introduction

“Advances are made by answering questions; discoveries are made by questioning answers” (Bernhard Haisch 1975) [1].

**Figure 1:**
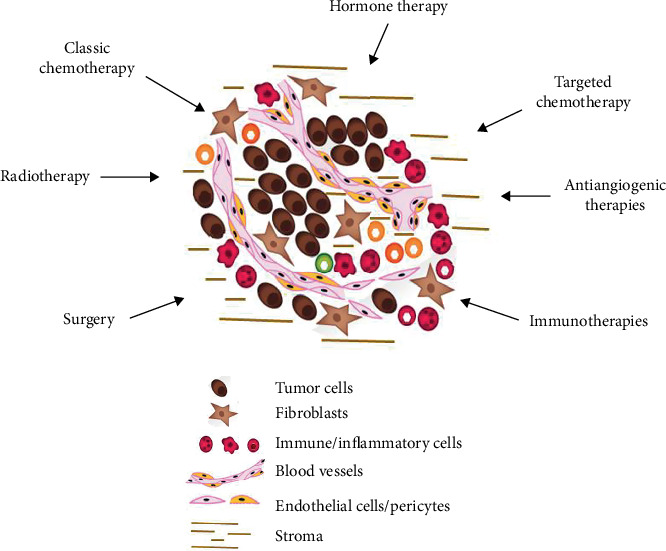
Therapeutic approaches currently used in cancer treatment. According to the “tumor-centric” paradigm, a cancer cure is only achieved after the complete eradication of the tumor burden.

The ultimate goal of all medical activity is to preserve health in fit people and to cure those who are sick, understanding by this as to restore a state of complete physical, mental, and social wellbeing on them. Our current therapeutic strategy is based on the fact that a cancer cure can only be achieved through the complete eradication of the tumor burden. Based on this and following the concept of the “poisoned arrow” described by Ehrlich, finding the “Achilles heel” of tumors that may be exploited as a specific target while sparing healthy tissues becomes essential [2]. At present, these vulnerabilities are being targeted by local and systemic treatments (Figure 1). This strategy has led to a substantial improvement in survival rates for cancer patients in an unprecedented way [3]. Despite this progress, more than 9 million people still die from cancer every year [4]. In addition, cancer survivors suffer chronic morbidities that impair their quality of life [5, 6]. We must then admit that, in most patients, the “poisoned arrow” strategy (mutated into “magic bullet”) is not leading to a cancer cure, that is, restoring cancer patients to a state of complete physical, mental, and social wellbeing, but to a cancer remission, that is, “the temporary absence of manifestations of a particular disease” [7, 8]. Certainly, current treatments prolong the life of cancer patients and improve their quality of life. We cannot vilify these impacts on every patient life. Any additional time gained with the current treatments can mean a lot to a patient with the prospect of dying. However, inducing a remission is not the same as curing cancer. After months or years of remission, cancer will inevitably recur. We can continue looking for other vulnerabilities in tumors, but the problem will persist.

Therefore, the factual question remains unanswered: how can we truly cure cancer? We can only find the answer to that question if we first accept that our current cancer treatment paradigm is obsolete. The evaluation of the present paradigm shows many triumphs in basic and clinical research but, unfortunately, continues to fail in our goal of restoring a state of complete physical, mental, and social wellbeing in most cancer patients. To cure the approximately 18 million people with cancer worldwide, we must shift from this paradigm [4]. It is time to pause and think about the key challenges and future directions in clinical oncology. What do we need to do in order to create a new kind of collective intelligence to truly cure cancer? How can we make cancer research smarter? Patients who are dying from cancer cannot wait. There is already enough experimental and clinical evidence to generate a new paradigm based on novel axioms that would allow us to achieve a cure, and not just a remission. This new paradigm would ensure that the right care is delivered to the right patient at the right time. Herein, we describe what we believe should be the axioms of this new cancer paradigm and how it may help to attain our objective of truly curing cancer patients.

## 2. A Need to Start from Scratch: Lessons from Hippocrates

“Physician must convert or insert wisdom to medicine and medicine to wisdom” (Hippocrates) [9].

Modern medicine is based on the works of Hippocrates (460-370 BC) and his disciples [9, 10]. In essence, Hippocrates claimed that any disease is based on natural causes, and therefore, in order to establish a diagnosis, a prognosis, and a treatment, medicine should be based on detailed observation, reason, and experience. This Hippocratic approach allowed a broader understanding of the causes, context, and clinical course of a particular disease. It is remarkable how much Hippocrates' work can be relevant today. Hippocrates taught us two main lessons: (i) cancer is a systemic (“humoral”) disease, i.e., a disease that affects the whole body, and not just a specific organ; (ii) a cancer cure can only be achieved by rebalancing the whole organism through a multidisciplinary, holistic approach, and not just by eradicating the tumor.

## 3. A New Cancer Paradigm: “For Systemic Diseases, Systemic Methods of Cure”

The aim of cancer treatment should be to restore a state of complete physical, mental, and social wellbeing in cancer patients, and not only to eradicate the tumor burden. Then, we need to move from the paradigm that claims “for extreme diseases, extreme methods of cure” to a new paradigm that may be reformulated as “for systemic diseases, systemic methods of cure”. This new paradigm should be based in new axioms that will lead to novel treatment strategies (Table 1).

### 3.1. Axiom #1: “The Tumor as an Organ-Like Structure, i.e., Another Member of a Complex Organism”

“The exacerbations and remissions will be indicated by the diseases, the seasons of the year, the reciprocation of the periods, whether they occur every day, every alternate day, or after a longer period, and by the supervening symptoms” (Hippocrates) [11].

An important breakthrough in cancer biology was the recognition of tumors as abnormal organs acting in the context of an entire organism [12–17]. Tumor metabolism is not necessarily autonomous or self-perpetuating. Tumor nourishment and growth are sustained by the interaction of the tumor with its host. Hormone-dependent breast and prostate cancers constitute examples of the direct interplay between the abnormal organ (tumor) and the organism as a whole. Furthermore, tumors also interact with nearby organs or surrounding tissues, as well as other organs, via glycolysis by-products generated by the specific metabolism of tumors, inflammatory tumor-released cytokines, and other poorly defined circulating components that constitute what is known as the “tumor-derived macroenvironment” [18–20]. Patient mortality is more related to these tumor-induced systemic alterations than to the direct effect of the primary tumor itself or even metastases.

A clear effect of the tumor-derived macroenvironment is the severe suppression of the immune system, resulting in an increased risk of infections and patient mortality [21, 22]. Tumors also activate procoagulatory factors and inhibit fibrinolytic factors, leading to the development of thrombosis, a major complication in cancer patients [23]. Indeed, thromboembolism is estimated to be the second most common cause of cancer-related mortality [24]. In addition, tumor-secreted factors (e.g., TNF*α*, IFN*γ*, IL6, and lactate) can disrupt the metabolic functions of the liver and lead to cachexia, which accounts for nearly one-third of cancer deaths [25, 26]. Furthermore, the heightened energetic demand of cancer cells can lead to substantial alterations in hepatic circadian metabolism, with altered insulin levels, glucose intolerance, and deregulated lipid metabolism [20]. Given that these metabolic effects are systemic, it is most likely that tumor-induced metabolic rewiring may also take place in multiple organs, disrupting homeostasis [27, 28]. All these highlight the myriad of complex systemic effects that a tumor can have on its host. As described by Rudolph Virchow (1821-1902), the life of an organism is based on collective features, not individual ones [29]. These features include “abnormal organs” such as tumors, supporting the notion of cancer as a systemic disease: tumors regulate and are regulated by processes that occur both inside and outside the local tumor microenvironment.

### 3.2. Axiom #2: “Concomitant Resistance (or Game of Thrones)”

“What remains in diseases after the crisis is apt to produce relapses” (Hippocrates).

The “societal” organization of cancer may be compared to the behavior of certain species of ants, which form complex organizations known as supercolonies, with the ability to expand their nests over large areas allowing them to achieve ecosystem dominance [30, 31]. Indeed, cancer multiple sites in a patient can be considered as supercolonies; however, unlike ants, these cancer colonies fight against each other for the “iron throne.” This phenomenon, first described by Ehrlich and other contemporary investigators, is known as “concomitant resistance” and describes a biological situation in which, upon certain circumstances, a tumor exerts a controlling and inhibitory action on other concomitant tumors, while paradoxically, it continues to grow [32–34]. A similar situation may happen among the different metastatic *foci*, where a main or “leading” metastasis inhibits the growth of the other metastatic colonies. Nowadays, we know that there are four main mechanisms of concomitant resistance: (i) the growth of a tumor might generate a specific antitumor immune response, which even though not strong enough to inhibit its growth, it is capable of preventing the development of small secondary tumors [35, 36]; (ii) tumors produce antiangiogenic molecules that suppress the vascularization and growth of small metastases [37]; (iii) the “athrepsia theory,” according to which essential nutrients for tumor growth are mostly consumed by the “main” tumor, makes it difficult for secondary tumors in other sites to grow [32]; (iv) the high metabolic rate of tumors induces the release into the bloodstream of metabolic by-products that further induce a state of dormancy of cancer cells in small metastatic sites. Meanwhile, the primary tumor or main metastasis is protected from their inhibitory effect due to the presence of counteracting amino acids and, therefore, continues to grow [32, 38, 39].

The concomitant resistance model explains the patterns observed in the clinical evolution of some types of cancers. For example, disease recurrence in patients with early breast cancer shows a bimodal pattern, with a broad dominant early peak of relapse at about 1.5-2 years after surgery, followed by a second peak at about 5 years, and then a tapered pattern of relapse extending up to 20 years [40, 41]. According to this evidence, the first peak of relapse is predominantly the result of surgery-promoted growth of dormant micrometastases due to the annihilative effects of surgery on concomitant resistance, while the second peak of relapse is explained by the natural stochastic transitions from dormant to active states [42, 43]. Wound healing and local inflammation induced by surgery might also promote the growth of micrometastases, further enhanced by surgical stress-induced immunosuppression, which helps cancer cells to circumvent immune-mediated rejection at micrometastatic foci [44, 45]. This clinical evolution has also been observed in patients who undergo surgery for primary control of prostate, lung, and pancreatic cancers, as well as osteosarcoma and melanoma [46–50]. A similar situation may develop among the different metastatic foci, where a main or leading metastasis inhibits the growth of other metastatic colonies. For example, liver metastases reappear within the first 2 years in 70% of colorectal cancer patients that underwent resection of liver-limited metastases [51, 52]. Likewise, most cancer patients with multiple metastatic sites who receive treatment with systemic therapies achieve complete or partial responses on a specific site, and after a short period of time, the disease returns in other sites.

### 3.3. Axiom #3: Chronic Inflammation—“the Source of All Evil”

One of the first researchers to link cancer and inflammation was Virchow, who suggested that irritation of the stroma caused sites of “chronic inflammation” with tumorigenic potential [53]. Years later, Theodor Boveri (1862-1915) provided evidence to support Virchow's ideas, suggesting that tissue inflammation induced chromosomal abnormalities during mitosis, while providing the environmental conditions required by this cancer cells to divide and proliferate [54, 55]. Virchow and Boveri were both right: inflammation promotes tumorigenesis through complex processes that lead to the transformation of healthy cells into cancer cells [56, 57]. Specifically, if inflammation becomes chronic, neutrophils and macrophages remain permanently in the affected tissue, producing reactive oxygen species (ROS) and reactive nitrogen species (RNS), proangiogenic factors (e.g., VEGF), cell-growth-promoting factors (e.g., IL1 and IL6), and immunosuppressive and profibrotic factors (e.g., TGF*β*) [58, 59]. ROS and RNS can damage DNA, increasing the frequency of random cancer-inducing mutations [12, 60]. The resulting genetic events can then activate the production of proinflammatory mediators, and the recruitment or activation of more inflammatory cells, thus perpetuating chronic inflammation [61]. Over time, the chronically inflamed tissue may become a cancerous tissue. Once the tumor is established, this vicious circle is closed with the maintenance of chronic inflammation through the constant release of proinflammatory factors by malignant cells [62]. Therefore, carcinogenesis can be seen as the perpetuation of unresolved local inflammation.

#### 3.3.1. The Unseen Enemy: Systemic, Low-Grade Chronic Inflammation

Alternatively, recent evidence suggests that cancer may also be the result of systemic, low-grade chronic inflammation (SLGCI). In SLGCI, peripheral tissues chronically exhibit high levels of inflammatory factors (C-reactive protein or CRP, TNF*α*, IL1*β*, IL6, and IL17) and infiltrated immune cells (macrophages, neutrophils, and T-lymphocytes) without exhibiting structural alterations or loss in their primary functions [63, 64]. It is worth noting that SLGCI plays a fundamental role in the pathogenesis of several noncommunicable and putatively unrelated chronic diseases [65–67]. Moreover, a vicious cycle of sustained SLGCI has been observed in some health conditions of virtually all organs, in parallel with the global adoption of modern environmental and lifestyle changes [67, 68].

The white or visceral adipose tissue plays a role in one of the primary mechanisms involved in the onset of SLGCI. The visceral adipose tissue secretes a wide variety of cytokines and other mediators in order to regulate specific metabolic, endocrine, and immune functions to maintain whole-body homeostasis [69]. Chronic overeating induces severe alterations to this regulatory system. When caloric intake exceeds energy expenditure, insulin levels raise, signaling adipocytes to take up glucose and convert it into an excess of triglycerides and fatty acids, leading to adipocyte hyperplasia and hypertrophy. This induces hypoxia and the subsequent necrosis of white adipose tissue, which is invaded by macrophages, which switch to a proinflammatory phenotype, thereby promoting local inflammation [70, 71]. This tissue damage and the ensuing inflammation promote cellular proliferation via an influx of other immune cells (e.g., neutrophils), proinflammatory mediators (leptin, IL6, Csf1, etc.) and growth factors, tissue remodeling, and angiogenesis. The large amount of fatty acids stored in adipocytes also exacerbates lipoperoxidation, with a considerable increase in ROS and RNS levels. This oxidative burst increases the recruitment of immune cells to the adipose tissue, further promoting inflammation [72]. By contrast, hypertrophic adipocytes reduce the production of anti-inflammatory or insulin-sensitizing factors, such as adiponectin [73]. Furthermore, excess free fatty acids can enter the systemic circulation, reaching other distant organs (e.g., muscle and liver), where they interact with toll-like receptors (TLRs), activate innate immune cells, and initiate the production of proinflammatory mediators (IL6 and MCP1), ultimately bringing inflammation to a systemic level [74, 75]. Altered metabolic states such as dyslipidemia and hyperglycemia can also induce an inflammatory response by activating macrophages through TLRs, which could later reach a systemic level when these cells migrate to insulin-dependent tissues and alter the micro- and macroenvironments of the body through increased cytokine release.

It is possible that, as in obesity, diabetes, and cardiovascular disorders, the unspecific activation of the immune system or SLGCI may also constitute an important risk factor for cancer [76, 77]. Indeed, obesity, overweightness, and diabetes increase cancer risk as well as the likelihood of death from certain types of cancer [78–80]. A clear example of how obesity-related inflammation can induce cancer is pancreatic-ductal adenocarcinoma. In a healthy pancreas, obesity promotes steatosis, inflammation, and fibrosis by modeling a specific microenvironment characterized by the accumulation of hypertrophic adipocytes, which secrete high amounts of cytokines, such as IL1*β* [81]. This promotes stellate cell activation, increased desmoplasia, neutrophil infiltration, and inflammation, thus promoting tumorigenesis [82]. Once established, malignant cells coopt the inflammatory mechanisms responsible for tissue repair to promote tumor growth and invasion.

#### 3.3.2. The Revenge of the Nerds: The Innate Immune System

Therefore, rather than being a passive reaction to cancer cells, inflammation seems to play a key active role in carcinogenesis. Innate immune cells, which were always considered as the “nerds” of tumor immunity, are largely responsible for these inflammatory reactions. A common characteristic of innate immune cells is their great phenotypic plasticity. This versatility makes these cells capable of displaying different functions regulated by signaling. For instance, during the early phases of inflammation, these cells will primarily promote the activation of the adaptive immune system, while in the chronic stage, they will suppress the immune reaction and promote tissue repair by activating angiogenesis and initiating stromal generation [83, 84]. Myeloid-derived cells are the main components of the innate immune system and are mainly divided into mononuclear and polymorphonuclear cells [12]. Mononuclear phagocytes include macrophages, which reside in virtually all tissues [85, 86]. Although it is acknowledged that there is a spectrum of intermediate states, macrophages present two major distinct phenotypes: M1, which promotes inflammation, and M2, which suppresses it. On the other hand, polymorphonuclear phagocytes or granulocytes are mainly neutrophils that accumulate in sites of inflammation and disease [87, 88]. Neutrophils can also be divided into two major distinct phenotypes: N1 and N2, which are pro- and anti-inflammatory, respectively [89, 90]. Special consideration should be given to MDSCs, which broadly include immature myeloid progenitors, and monocyte- and granulocyte-like cells, and are characterized by their immunosuppressive ability [90–92].

#### 3.3.3. An Inside Job: Role of the Immune Cells in Tumors

Inflammatory cells are also major cellular components of established tumors [83, 84]. Tumor-associated macrophages (TAMs) are derived from monocytes that are recruited into tumors by chemokines secreted by both malignant and stromal cells. Once in the tumor, TAMs are “conditioned” by the tumor microenvironment towards switching to an M2-like state, in order to display a number of protumoral functions such as promotion of tumor cell proliferation and survival, induction of immunosuppression and angiogenesis, and matrix remodeling and metastasis [84, 93]. Similarly, tumor-associated neutrophils (TANs) are conditioned into N2-like neutrophils promoting tumor formation by producing ROS and RNS species, tumor proliferation factors (e.g., neutrophil elastase), angiogenic factors (e.g., VEGFA), and ECM-degrading enzymes (e.g., MMP9). N2 neutrophils also suppress antitumor immune response by the release of inducible nitric oxide synthase (iNOS), arginase 1, or TGF*β* [94, 95]. These functions can be exerted locally, in or around the tumor microenvironment, as well as systemically, in distant organs. The role in cancer of other granulocytes, such as mast cells, eosinophils, and basophils, remains lesser known [85, 96, 97].

#### 3.3.4. Friends as Foes: The Microbiota

Another source of SLGCI is the microbiota [98, 99]. Any imbalance of the microbiota (dysbiosis) may disrupt the symbiotic relationship with its host organism, promoting inflammation and diseases such as hypertension, diabetes, or obesity [100, 101]. Dysbiosis *per se* is also a source of chronic inflammation and carcinogenesis [102, 103]. For example, metagenome-wide association studies in stool samples from patients with colorectal cancer suggest that harmful bacteria in the gut may become more abundant in response to unhealthy lifestyle or deleterious dietary habits. This increases the exposure of the gut epithelium to potentially mutagenic metabolites. On the other hand, the metabolites produced by, for example, fruit and vegetable consumption facilitate the maintenance of the colonic epithelium through inducing a relatively low pH, which helps to reduce amino acid fermentation and pathogen growth. Therefore, depleting these protective metabolites may also promote the development of colon cancer [101, 104, 105].

### 3.4. Axiom #4: “Augmented Intelligence—the Doctor's Sixth Sense”

“One, then, ought to look to the country, the season, the age, and the diseases in which they are proper or not” (Hippocrates).

**Figure 2:**
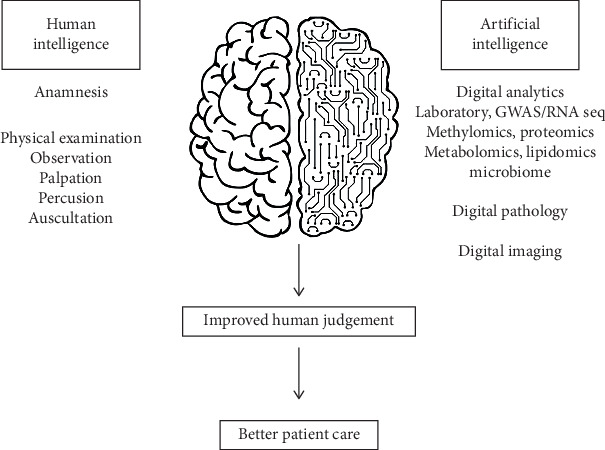
A new collective intelligence for a smarter patient care. Artificial intelligence should be viewed as a complementary tool for the physician. With the help of artificial intelligence systems, oncologists will be able to combine its clinical knowledge with massive amounts of data. As a consequence, oncologists will be able to discern new relevant patterns and will gather insights in a way that human intelligence alone cannot do, ultimately improving diagnosis and treatment.

Treatment decisions are often based on data obtained from a small percentage of patients, i.e., those who take part in clinical trials. This means that clinical information from the majority of patients, who do not take part in clinical trials, is not being used [106]. This is changing with the emergence of artificial intelligence (AI) technologies, which have the ability to work with enormous amounts of data. But AI generates many fears and apocalyptic thoughts due to its potential to replace humans in many different tasks. However, these fears are unjustified, and AI should be viewed as a tool to complement the physician's work, not as a replacement. Then, what can AI add to our human intelligence in healthcare settings? Nowadays, the healthcare world is awash with vast, valuable sources of information. Indeed, improvements in mobile phone technology, sensors, and connectivity generate extraordinarily detailed insights into an individual's health status. AI can assimilate this massive amount of data, while discerning relevant patterns and insights that human intelligence is not capable to detect, allowing the application of real-world healthcare data to an individual's particular healthcare situation. However, AI technology is based on mathematic algorithms that do not have a physician's ability to see the big picture or take into consideration less quantifiable factors that affect a patient's health, let alone to be a substitute for human judgment. Thus, in the coming decades, the traditional role of the physician will be assisted by AI, helping to establish accurate diagnosis and reach wisertreatment decisions (Figure 2).

One of the most interesting areas of research in AI is virtual medicine. The human body is the biggest data platform. A variety of fields have used computational models as virtual surrogates of human physiology. These models can be considered as virtual representations of a subset or the whole of the patient's physiological identity [107]. Patient's data would be made available to the virtual model through integration of electronic health records (EHRs), mobile health (mHealth) devices, telemedicine, electronic patient-reported outcomes (ePROs), and other platforms, tools, or media [108]. Available patient information should include medical history, genetic idiosyncrasies, environmental factors, diet, lifestyle, particular behaviors, preferences, socioeconomic status, location, data recorded by wearable sensors and mHealth devices, and access and adherence to treatment recommendations. MRI and CT scans can also be used to produce a virtual geometric and physiologic view of the patient, reproducing individual anatomy, organ structure, and temporary blood flow. These virtual representations of each individual patient can then be used to tailor prevention, diagnosis, therapeutics, and prognosis for each individual patient [109, 110].

## 4. Novel Treatment Strategies

It is clear that a cancer cure will not be achieved only by the eradication of the tumor. Indeed, a tumor-centric treatment can never be a solution to a systemic, medical problem. With this in mind, how can we treat cancer patients to truly cure them? We can start by following the example of other therapeutic areas. The clearest is the paradigm shift produced in the treatment of the metabolic syndrome, a cluster of conditions comprising increased blood pressure, high blood sugar, excess body fat around the waist, and increased cholesterol or triglyceride levels. In the past, all these conditions were diagnosed and treated separately, without a global approach. Today, we know that all these markers are related to chronic inflammation, tend to occur together, and increase the risk of heart disease, stroke, and diabetes. Indeed, once established, SLGCI promotes and perpetuates metabolic alterations, establishing a deleterious cycle that promotes pathological processes such as insulin resistance, arteriosclerosis, and endothelial dysfunction. In order to break this cycle, it is necessary to control both the metabolic and inflammatory components simultaneously. These new concepts led to a shift to therapeutic strategies with a more holistic, multidisciplinary approach [111]. Firstly, a person diagnosed with metabolic syndrome or any of its components needs some radical lifestyle changes. A lifelong commitment to a healthy lifestyle includes actions such as being physically active, losing weight, eating a healthy diet, stop smoking, and managing stress. Only with these measures can the development of diabetes or cardiovascular disease be delayed or prevented. If implementing lifestyle changes is not enough, pharmacological treatments to control blood pressure, cholesterol levels, and blood glucose may also be applied. A similar approach should be used for cancer prevention and treatment, adding to the current strategies, new treatment modalities based on the four axioms that define the new cancer treatment paradigm.

### 4.1. Evaluation of a Person's Health Status on a Regular Basis

As an old medical adage goes, it is better to prevent a disease than to treat it. This is why the first important step is to educate people so that they can take care of their own health. Simultaneously, primary care should not only include a global clinical analysis and routine imaging/laboratory tests but also include the assessment of SLGCI markers on a regular basis and the characterization of the patient's microbiota whenever possible. The routine analysis of these variables by clinical, imaging, and laboratory tests will allow to determine the levels of chronic inflammation. Therefore, those identified as having a high risk of cancer (among other diseases) based on their level of chronic inflammation can receive early intervention strategies.

### 4.2. “Mens Sana in Corpore Sano [112]”: Lifestyle Changes for a Holistic Approach to Wellbeing

As with other chronic diseases, cancer is linked to unhealthy lifestyles such as smoking, poor diet, lack of physical activity, obesity, sleep deprivation, and alcohol abuse, along with the daily chronic stress imposed by modern life. Thus, current global cancer prevention and treatment strategies should firstly focus on drastically modifying people's lifestyles. Indeed, individuals with a healthy lifestyle appear to have a remarkably lower risk of cancer [113, 114]. This is an enormous social challenge because it involves forcing people to confront their habits, attitudes, and behaviors. In addition to quitting smoking or reducing alcohol intake, there are some basic measures that everybody can apply with impressive results.

#### 4.2.1. Daily Physical Activity

Physical activity induces a series of adaptive processes that affect tissue metabolism, angiogenesis, and immune regulation [115]. For example, physical activity triggers an intramuscular, inflammatory immune response which induces macrophage differentiation towards a proinflammatory M1 phenotype [116, 117]. Regular physical activity markedly reduces the risk of the primary development of several cancers and might improve clinical outcomes following the diagnosis of a primary tumor [118, 119]. Similarly, there is increasing evidence that supports the role of physical activity in cancer treatment, and it is well accepted that structured, regular physical activity is feasible and well-tolerated in cancer patients [120–122]. Physical activity for as little as 15 minutes a day is associated with a 10% reduced risk in cancer mortality, and every additional 15 minutes of daily physical activity beyond this is associated with an additional 1% reduction in risk [123]. Nevertheless, it is important to stress that response to physical activity prescription is considerably heterogeneous among individual patients, and thus, it should be appropriately prescribed on a case by case basis [124–126].

#### 4.2.2. Healthy Diet

Different studies have consistently shown a correlation between increased body weight and cancer, with increased mortality rates for all cancers and for cancers at multiple specific sites in obese patients [127–129]. Importantly, adipose-related inflammation and its associated tumorigenic effects have also been observed in one-third of individuals who are not considered to be obese or overweight based on their body mass index [130]. Several studies provide strong evidence that caloric restriction inhibits carcinogenesis [131, 132]. It is calculated that more than 90,000 cancer deaths per year might be avoided if the adult population could maintain a body mass index below 25 throughout their entire life [133–135]. This evidence supports the development of interventions that reduce adipose-related inflammation as a new strategy for cancer prevention and treatment. A modest reduction in fat intake with minimal weight loss represents an easily achievable goal to reduce cancer mortality.

#### 4.2.3. Maintaining a Natural Sleep-Wake Cycle

Sleep has an impact on a vast array of physiological functions, such as immune function, cognitive ability, and glucose metabolism [27]. When sleep is disturbed or restricted, all these physiological processes are affected. For example, lack of sleep shortness can result in significant changes in a number of circulating proinflammatory cytokine (e.g., IL6 and IL1*β*) and cortisol levels [136–138]. The effects of sleep deprivation are cumulative. Therefore, over a period of time, sleep debt can lead to a wide range of deleterious health consequences, including SLGCI [139–141]. Sleep shortness can be solved with simple steps (going to sleep and getting up at the same time every day, controlling exposure to light before and during sleep, improving the sleep environment, etc.).

### 4.3. Interventional Measures

Once a primary tumor is diagnosed, the therapeutic strategy must be adapted to each patient: this is the only true personalization of medicine. Firstly, we must have new diagnostic tests to evaluate, besides tumor genetics and TME, the degree of interrelation of the tumor with its host organism (e.g., other organs or microbiome), its micro- or macrometastases (concomitant resistance), and the degree of SLGCI. Then, a global approach taking into consideration all these variables must be adopted. For example, we have previously discussed how obesity stimulates pancreatic tumor initiation, growth, and metastasis [142–144]. Thus, the correct strategy to treat this type of patients would be to target not only the tumor but also its original cause, i.e., SLGCI due to obesity. This holistic strategy should include physical activity and diet to reduce obesity and restore normobiosis, combined with treatments to deplete TANs, inactivate PSCs, and inhibit IL1*β* secretion, so as to prevent obesity-promoted tumor growth and desmoplasia. In preclinical models, this strategy is proved to be successful [143].

#### 4.3.1. Maintenance of the Concomitant Resistance Phenotype

Novel therapeutic strategies should modulate specific tumor microenvironments to maintain them in a dormant state. Current adjuvant chemotherapy should be administered with different schedules that could be more effective than standard regimens. For example, in surgically treated breast cancer patients, first recurrences tend to occur after 1.5-2 years. Thus, it may be worth to reintroduce specific systemic treatments during that time period in order to eradicate the growing micrometastatic foci; in this sense, the use of oral metronomic chemotherapy appears to be the most interesting schedule as, besides showing antitumor activity, it modulates tumor angiogenesis and immune responses with a moderate toxicity [91, 145]. Oral metronomic chemotherapy should also be used 4-5 years after surgery to prevent the second relapse surge. On the other hand, the use of drugs that alter metabolic stress in cancer cells could be an interesting alternative to modulate the metabolic balance between the primary tumor and its metastases [146]. This could be combined with the administration of metabolic by-products such as *m-*Tyr. This metabolite was recently shown to exert its antimetastatic effect at low concentrations with no detectable toxic side effects [147]. Surgical stress, besides disrupting concomitant resistance, and inducing immunosuppression and inflammation, also awakens micrometastatic foci from their dormant state [44, 148]. Excessive surgical stress and postoperative complications cause a storm of perioperative cytokines, which has been shown to promote tumor metastasis [149]. Thus, anti-inflammatory drugs may potentially avoid the undesirable effects of surgery. It is interesting to note that some publications showed that in surgically treated breast cancer patients, perisurgical administration of ketorolac dramatically reduced first-peak recurrences [150, 151]. Minimally invasive surgical techniques should also be used to lessen surgical stress.

#### 4.3.2. Treatment with Anti-Inflammatory Drugs

Therapeutic modulation of chronic inflammation is likely to transform a protumorigenic microenvironment into a healthy microenvironment. For this reason, several commonly used medications, including nonsteroidal anti-inflammatory drugs (NSAIDs), metformin, and statins, represent an additional tool in cancer treatment. For example, COX2 inhibitors are effective cancer chemopreventive agents and have also demonstrated to control cancer-associated chronic inflammation [152, 153]. Effectively counteracting or neutralizing tumor-promoting chronic inflammation can also be achieved by the simultaneous reprogramming of multiple immune response pathways that are activated in cancer. On the basis of the available data, the pathways that currently represent attractive targets for cancer treatment include: (i) trafficking inhibition, depletion, or reprogramming of tumor-associated innate immune cells and (ii) inhibition or sequestration of cytokines or chemokines. All these strategies have an impact on nonredundant mechanisms, and hence, they may be more successful in combination.

#### 4.3.3. Restoration of a Healthy Microbiota

Manipulation of the gut microbiome to restore a protective microbiota (eubiosis) is a goal in patients that show disturbances in microbial composition and functionality (Raman 2013). Diet is the easiest way to restore eubiosis. For example, the consumption of fruits and vegetables promotes the growth of bacterial species that produce butyrate and lactate, which helps to preserve the colonic epithelium through inducing a relatively low pH. This might reduce amino acid fermentation and the growth of cancer-related pathogens [101, 104, 154]. In addition to diet, prebiotics, probiotics, or microbiota transplants may also be used to restore eubiosis, thereby reducing microbially induced genotoxicity and activation of inflammatory, proliferative, and antiapoptotic pathways. Prebiotics are defined as nondigestible substances that produce beneficial physiological effects on the host by stimulating, in a selective manner, the growth and metabolic activity of a limited number of beneficial indigenous bacteria while probiotics are live microorganisms that confer a health benefit on the host. Both prebiotics and probiotics can be used to prevent the onset of dysbiosis when the patient is exposed to predisposing conditions and as therapeutic agents to rebalance an ongoing dysbiosis [155]. Prebiotics act mainly as a specific fuel that indigenous probiotic bacteria can utilize to grow. Most commonly known prebiotics include fructo-oligosaccharide supplements, galacto-oligosaccharides, inulin, lactulose, and breast milk oligosaccharides. Instead, probiotic effects can be categorized as immunological and nonimmunological [156]. Immunological benefits include the activation of local macrophages, an increase in the production of immunoglobulin, the modulation of cytokine profiles, and the induction of hyporesponse to food antigens. Nonimmunological benefits include the digestion process, competition with potential pathogens for nutrients, and intestinal adhesion sites, pH alterations, and bacteriocins production. Currently used probiotics include lactic acid bacteria, bifidobacteria, enterococci, Saccharomyces boulardii, Bacillus spp., and propionibacterias. Finally, fecal microbiota transplantation is the most direct way to change the composition of gut microbiota. This treatment modality can have direct or indirect effects. Different series and case reports have revealed that fecal microbiota transplantation can alleviate various digestive (hepatocarcinoma, gastrointestinal, and pancreatic) and nondigestive (breast and melanoma) cancers linked to intestinal dysbiosis [157]. Additionally, it can be used to counter treatment-associated complications such as radiation enteritis, *C. difficile* infection, or graft-versus-host disease.

## 5. Back to the Future: Concluding Remarks

There is no doubt that throughout history, the current paradigm has significantly improved cancer care. In addition, nowadays, thanks to recent medical discoveries, patients with cancer live longer and with better quality of life than in the past. But the goal of curing all cancers has not been accomplished yet. We believe that the current paradigm regarding cancer treatment needs significant changes and considerable efforts. Currently, cancer therapy is still based on the millenary paradigm that establishes that in order to achieve a cure, the complete eradication of cancer cells must be achieved. The old philosophical concept of “magister dixit” (said by the teacher, then it is true) has encouraged the faithful fulfillment of the current paradigm despite the fact that, for a long time, no significant improvements have been observed in actual cure (not just remissions) rates, and despite recent scientific and technological advances. Consequently, should we be surprised by this despite the efforts invested in drug discovery and development, pharmacology, and technical devices? Probably not. What should be surprising is the insistence in maintaining a paradigm and axioms that do not work. Nowadays, what seems to matter is not what the medical community knows or does not know regarding a certain fact but rather what it believes or does not believe about it. The real individualization of cancer treatment consists in treating each individual patient following the good general practices of oncology and taking into consideration his/her own particular needs.

Our past reductionist approaches have led us to miss the bigger picture, look in the wrong places, or worse still, not even question if we have the right starting point [158]. According to the Hippocratic view, any treatment modality should consider the patient as a unique physical, mental, and social entity [159]. We need to regain the wisdom of Hippocrates. It is necessary to integrate genetic, biological, clinical, psychological, and social information into a new coherent framework or paradigm to transform it into knowledge and wisdom applied to the clinic that would lead to restoring cancer patients to their fullest physical, emotional, and social capacities. The new paradigm for cancer treatment should be based on this holistic view. As the old masters of medicine said, we must treat patients, not illnesses. We must move away from the “disease” silos and become patient focused [6]. We can start by taking the example of other therapeutic areas. As explained above, the clearest example is the paradigm shift produced in the understanding and treatment of the metabolic syndrome. A similar strategy based on a paradigm shift should be adopted in oncology. Behavioral approaches to control counterproductive lifestyles should be the first measure to prevent or treat cancer [160, 161]. Lifestyle changes do not have to be radical. For instance, simple measures such as eating wholefoods and a plant-based diet, stress management techniques, moderate physical activity, and having social support and being part of a community can slower the progression of localized prostate cancer [162]. These behavioral changes may be combined with pharmacological interventions to reduce SLGCI and the tumor burden.

Big data and AI are redefining cancer research and management [163, 164]. There are many opportunities of how AI can expedite the delivery of new therapeutic options to patients: from computational frameworks that match chemical identity to gene-based descriptors of disease mechanism, to a more unambiguous approach with distributive clinical trials that brings trials to patients (instead of patients to trials). On the other hand, the ability to generate virtual patients from research and clinical data would help to discover and develop novel transformative drugs adapted to the new axioms supporting the innovative paradigm. It should be noted that factual sciences are divided into natural sciences and social sciences. Medicine occupies a special and borderline place between both, and it is very difficult to establish the similarities between a doctor who works through established rules in clinical trials and a family practitioner. The first behavior will be more related to the natural sciences and the latter will be more related to the social sciences, what is known as “the art of medicine.” The combination of human and artificial intelligence in a new kind of collective intelligence would allow doctors to incorporate features of the two, i.e., to exercise the art of medicine (social sciences) based on data analysis (natural sciences).

In conclusion, cancer should not be seen as an invading organism or a mere cluster of cancer cells that needs to be eradicated. Instead, a tumor may be better understood as an organ-like structure, acting in the context of a whole organism (a systemic disease) of an individual patient living in a particular social reality. Such a view is more appropriate for understanding complex systems, where some properties of the whole system cannot be inferred from the separate properties of its individual components. Indeed, if one regards the tumor as an organ-like structure within an organism that is a human being (the patient), it seems less outlandish that cancer may be related to homeostatic imbalance, inflammation, or concomitant resistance. These new concepts highlight the notion that even “transient” phenomena can have an impact with lifelong consequences, or in the words of chaos theory, “the ultimate outcome is exquisitely sensitive to the initial conditions.” In summary, the understanding of cancer disease based on holistic clinical and pathophysiological concepts will finally lead to an improvement in the health and wellbeing of cancer patients. They deserve nothing less.

## Figures and Tables

**Table 1:** The new cancer treatment paradigm: “for systemic diseases, systemic methods of cure”.

*Objective:* To restore a state of complete physical, mental, and social wellbeing in cancer patients
*Axioms* Tumors as organ-like structures, member of a complex organism Concomitant resistance Chronic inflammation Local tissue inflammation Systemic, low-grade inflammation Augmented intelligence
*Therapeutic approaches* Lifestyle changes Daily physical activity Healthy diet Adequate sleep/wake cycles Maintenance of concomitant resistance Use of anti-inflammatory drugs Restoration of eubiosis Reprogramming of tissue and tumor metabolism New schedules of conventional treatments (surgery, radiotherapy, chemotherapy, hormone-therapy, antiangiogenic agents, and immunotherapy)
